# Impact of Antibiotic Exposure on Growth and Biofilms Formation in *Aeromonas salmonicida* Subspecies Isolated from Atlantic Salmon (*Salmo salar*)

**DOI:** 10.3390/microorganisms13122863

**Published:** 2025-12-16

**Authors:** Dong Hwi Kim, Min Soo Joo, Se Rin Jang, Hee Jin Kim, Joon Gyu Min, Bo Hye Nam

**Affiliations:** Aquaculture Industry Research Division, East Sea Fisheries Research Institute, National Institute of Fisheries Science, Gangneung 25435, Republic of Korea; mykdh21@hanmail.net (D.H.K.); jangid90@naver.com (S.R.J.); ken0814@naver.com (H.J.K.); cdmin0621@korea.kr (J.G.M.); nambohye@korea.kr (B.H.N.)

**Keywords:** *Aeromonas salmonicida* subspecies, antibiotic exposure, biofilm formation, quorum sensing, sub-inhibitory concentration

## Abstract

*Aeromonas salmonicida* is a major pathogen in aquaculture, and its ability to form biofilms contributes significantly to antibiotic resistance and chronic infections. This study investigated the effects of four antibiotics—ampicillin, amoxicillin, oxytetracycline, and doxycycline—at various concentrations on bacterial growth, biofilm formation, and gene expression related to antibiotic resistance and quorum sensing (QS) in two subspecies: *A. salmonicida* subsp. *masoucida* (ASM) and *A. salmonicida* subsp. *salmonicida* (ASS). Bacterial isolates from Atlantic salmon were identified using 16S rRNA and *vapA* gene sequencing. Growth inhibition was more pronounced in ASS than ASM under high antibiotic concentrations. Conversely, sub-inhibitory concentrations (sub-MICs) enhanced biofilm formation in both subspecies, particularly in ASM. PCR results showed that *tetA* and *tetE* resistance genes were present only in ASM. qRT-PCR analysis revealed that expression of QS-related genes (*ahyI* and *ahyR*) was generally downregulated under tetracycline treatment, while *litR* expression varied across antibiotic conditions and strains. Some isolates showed increased *litR* expression alongside elevated biofilm formation, suggesting involvement of additional regulatory mechanisms. These results highlight the potential for sub-MIC antibiotic exposure to promote biofilm development and modulate gene expression, emphasizing the need for careful antibiotic use in aquaculture and providing insight into alternative pathogen control strategies.

## 1. Introduction

Bacteria employ various strategies to resist antibiotics, among which biofilm formation is a key mechanism. A biofilm is a structured microbial community that adheres to biotic or abiotic surfaces and is embedded within a self-produced extracellular matrix, primarily composed of extracellular polymeric substances (EPSs) [1,2]. EPSs restrict antibiotic diffusion and create a hypoxic environment within the biofilm, further diminishing the efficacy of antimicrobial agents [3,4]. Quorum sensing (QS), a bacterial communication system, regulates metabolic activity, promotes biofilm development, and enhances virulence [5]. Biofilms confer several advantages to bacteria, notably collective resistance that enables survival even under high concentrations of antibiotics [6]. It has been reported that bacteria within biofilms exhibit significantly higher expression of resistance-related genes than their planktonic counterparts, resulting in antibiotic susceptibility that is 10-to-1000-fold lower [2,7,8,9,10]. Various environmental factors have been shown to influence biofilm formation, including temperature, pH, nutrient concentration, and the presence of antibiotics [11,12]. While some antibiotics inhibit biofilm formation, others may paradoxically induce it by activating bacterial defense mechanisms [13]. This phenomenon is closely associated with bacterial adaptive responses and has been particularly observed under sub-minimum inhibitory concentration (sub-MIC) conditions, where sublethal antibiotic exposure enhances biofilm development [14]. Thus, antibiotic administration may not only fail to suppress bacterial growth but may also promote chronic infections by inducing biofilm formation [15]. Consequently, increasing attention has been directed toward alternative strategies for biofilm control beyond conventional antibiotics. These include quorum sensing inhibitors (QSIs), biofilm-degrading enzymes (e.g., dispersin B, DNase I), and nanomaterial-based antimicrobials (e.g., silver nanoparticles, chitosan nanoparticles), which aim to reduce bacterial pathogenicity while minimizing the emergence of antibiotic resistance [16,17,18].

The genus *Aeromonas* comprises ubiquitous bacteria inhabiting diverse aquatic environments, including freshwater, seawater, aquatic organisms, and sediments [19]. Among these, *Aeromonas salmonicida* is the causative agent of furunculosis, a disease responsible for substantial economic losses in the Atlantic salmon (*Salmo salar*) aquaculture industry. Initially isolated from brown trout (*Salmo trutta*), *A. salmonicida* is a non-motile, Gram-negative bacterium [20,21,22]. Based on phenotypic, biochemical, and morphological characteristics, this species is divided into five subspecies: *salmonicida*, *smithia*, *achromogenes*, *masoucida*, and *pectinolytica* [23,24]. Although once considered specific to salmonids, *A. salmonicida* has recently been reported to infect a broad range of freshwater and marine non-salmonid species, owing to its diverse virulence factors, including the A-layer [25,26,27]. In aquaculture, antibiotics such as oxolinic acid, erythromycin, and florfenicol have been widely used to control *A. salmonicida* infections. However, the prolonged use of these antimicrobials has led to the emergence of resistant strains, posing a significant threat to treatment efficacy, aquaculture sustainability, and public health [24,28]. Moreover, *A. salmonicida* demonstrates considerable tolerance to environmental stressors, a trait that may be further enhanced in biofilm-associated cells [26]. The A-layer, a paracrystalline surface protein array analogous to the S-layer, is encoded by the *vapA* gene and plays a key role in surface attachment, which is essential for biofilm formation [29]. Recent studies have indicated that exposure to certain antibiotics may enhance biofilm formation in *A. salmonicida*, emphasizing the need to better understand the link between antibiotic exposure and bacterial persistence [1]. A comprehensive understanding of the relationship between biofilm formation and antibiotic resistance in *A. salmonicida* is critical not only for effective infection management in aquaculture but also for mitigating the environmental dissemination of bacterial pathogens.

Tetracycline resistance in *Aeromonas* species is primarily mediated by genes such as *tetA*, *tetB*, *tetD*, *tetE*, and *tetM*, which encode efflux pumps or ribosomal protection proteins that decrease intracellular antibiotic concentrations [28]. These genes are often located on plasmids and other mobile genetic elements, promoting horizontal gene transfer among aquatic bacteria and contributing to the persistence of multidrug-resistant strains in aquaculture environments [22,28]. The quorum-sensing (QS) system in *A. salmonicida* is mainly regulated by the *ahyI* and *ahyR* genes, which synthesize and recognize N-acyl homoserine lactones (AHLs) to coordinate virulence factor secretion and biofilm formation [5,21,25]. In contrast, *litR* functions as a QS repressor, modulating AHL-mediated activation under stress conditions [5,26]. Understanding the interaction between antibiotic resistance and QS regulatory networks provides key insight into how *A. salmonicida* adapts to antibiotic stress and maintains persistence in aquaculture systems [1,2,6].

*A. salmonicida* employs various signaling and regulatory mechanisms to survive in host environments and establish infection. Among these, second messenger systems particularly cyclic di-GMP (c-di-GMP) play a pivotal role in the regulation of biofilm formation and virulence [30]. Elevated intracellular c-di-GMP levels promote surface attachment and biofilm development, whereas reduced levels favor a planktonic lifestyle [31]. A deeper understanding of the regulatory mechanisms governing biofilm formation in *A. salmonicida* could provide valuable insights for developing effective disease control strategies in aquaculture. In addition, host immune responses can significantly influence the biofilm-forming behavior of *A. salmonicida*. Host-derived factors such as mucus, immune proteins (e.g., lysozyme and components of the complement system), and oxidative stress (e.g., reactive oxygen species, ROS) have been shown to affect bacterial adhesion and survival strategies [32,33]. Several studies suggest that immune pressure exerted by the host may even stimulate biofilm formation, thereby contributing to the establishment of chronic infections [34]. Beyond the host, *A. salmonicida* also exhibits diverse ecological adaptation strategies that facilitate its survival and dissemination in aquatic environments. Previous research has demonstrated that interactions with symbiotic or coexisting microorganisms can enhance biofilm formation and influence antibiotic susceptibility [35]. These findings suggest that microbial interactions in natural ecosystems may further increase the environmental persistence and transmission potential of *A. salmonicida*.

The present study aims to analyze biofilm formation and growth responses to antibiotic treatment in *Aeromonas salmonicida* subspecies *masoucida* and *salmonicida* to elucidate the relationship between biofilm development and antibiotic resistance. Specifically, we investigate how different antibiotics and their concentrations influence biofilm formation in these subspecies, providing essential scientific evidence to inform effective infection management strategies.

## 2. Materials and Methods

### 2.1. Ethical Statement

All animal experiments conducted in this study received approval from the Institutional Animal Care and Use Committee of Jeju National University. This study was conducted strictly in accordance with the guidelines of the European Union directive 2010/EU. Furthermore, all experimental protocols were conducted by scientists who had completed animal ethics training.

### 2.2. Bacterial Strains

Bacterial analysis was conducted on Atlantic salmon reared in recirculating aquaculture systems (RASs) at two facilities located in Gangwon Special Self-Governing Province in 2023 and 2024. A total of 26 Atlantic salmon (16.86 ± 2.5 cm, 37.81 ± 20.4 g) collected in 2023 were reared in seawater at 13 °C. In 2024, 41 smolt-stage Atlantic salmon (33.72 ± 7.32 cm, 49.41 ± 44.2 g) were reared at 13 °C in water with a salinity of 5 psu. For bacterial isolation, samples were collected from clinical lesions, kidney, and spleen, then streaked on tryptic soy agar (TSA, Difco, Detroit, MI, USA) and incubated at 20 °C for 24–48 h. After incubation, presumptive *A. salmonicida* colonies were identified based on their distinctive morphology, appearing as smooth, convex, brown-pigmented colonies. Pure colonies were subsequently re-streaked and verified by PCR amplification of the 16S rRNA and *vapA* genes. After pure colonies were obtained, genomic DNA was extracted and PCR was performed.

### 2.3. Pathogen Identification

DNA was extracted from purified bacterial isolates using the AccuPrep^®^ Genomic DNA Extraction Kit (Bioneer, Seoul, Republic of Korea). Initial identification was conducted by sequencing the 16S rRNA gene, followed by subspecies identification through sequencing of the *vapA* gene. PCR was carried out using AccuPower^®^ PCR PreMix (Bioneer, Seoul, Republic of Korea). Primers for the 16S rRNA gene were based on ref. [36]: 27F (5′-GAGTTTGATCCTGGCTCAG-3′) and 1492R (5′-GGTTACCTTGTTACGACTT-3′). Primers for the *vapA* gene followed ref. [37]: forward (5′-TCAACGGATAGGTTCAACCC-3′) and reverse (5′-CAGAGTGAAATCTACCAGCGGTGC-3′). The PCR conditions for the 16S rRNA gene were: 94 °C for 5 min; 35 cycles of 94 °C for 30 s, 53 °C for 30 s, and 72 °C for 1 min; and a final extension at 72 °C for 7 min. For the *vapA* gene: 94 °C for 5 min; 35 cycles of 94 °C for 30 s, 54 °C for 30 s, and 72 °C for 30 s; and a final extension at 72 °C for 7 min. PCR products were analyzed by electrophoresis on 1.5% agarose gel and sequenced for identification using BLASTn (NCBI, accessed via the NCBI website).

### 2.4. Assessment of Growth and Biofilm Formation in Response to Antibiotic Concentrations

All antibiotics used in this study—ampicillin, amoxicillin, doxycycline, and oxytetracycline—were purchased from Sigma-Aldrich (St. Louis, MO, USA) as analytical-grade reagents (purity ≥ 98%, verified by the manufacturer’s certificate of analysis). Each compound was accurately weighed using an analytical balance (±0.1 mg) and dissolved in sterile distilled water (or methanol for doxycycline) to prepare stock solutions according to the manufacturer’s specifications. The concentration of each antibiotic was confirmed by accurately weighing the compound according to its molecular weight and solubility data, followed by serial dilution in CAMHBT broth. The prepared working solutions were validated spectrophotometrically based on their specific absorbance peaks (λmax) to ensure accuracy and reproducibility. To assess bacterial growth, pre-cultured bacterial cells were suspended in sterile distilled water and adjusted to a turbidity of 0.5 McFarland standard (1.5 × 10^8^ CFU/mL). The suspension was diluted in CAMHBT broth (cation-adjusted Mueller–Hinton broth with TES, TREK Diagnostic Systems Ltd., East Grinstead, UK) to a final concentration of 5 × 10^5^ CFU/mL. Four antibiotics were tested: ampicillin (ranging from 1.28 μg/mL to 128 mg/mL), amoxicillin (1.6 μg/mL to 160 mg/mL), oxytetracycline (1.28 μg/mL to 128 mg/mL), and doxycycline (0.64 μg/mL to 64 mg/mL). Bacterial suspensions (150 μL) were dispensed into 96-well plates (SPL, Pocheon, Republic of Korea), and 50 μL of each antibiotic at various concentrations was added. For controls, 150 μL of CAMHBT broth was used as the negative control, and 150 μL of the diluted bacterial suspension was used as the positive control. The plates were incubated at 20 °C for 24, 48, 72, and 96 h, and bacterial growth was assessed by measuring optical density at 600 nm (OD_600_).

For biofilm quantification, culture media were removed from the wells, which were then washed four times with sterile distilled water and air-dried. Each well was fixed by incubation with 200 μL of methanol (Sigma-Aldrich, Saint Louis, MO, USA) for 15 min and air-dried again. Biofilms were stained with 200 μL of 2% crystal violet (MB Cell, Seoul, Republic of Korea) for 5 min, washed twice with sterile distilled water, and dried. The bound dye was solubilized with 200 μL of ethanol (Sigma-Aldrich, Saint Louis, MO, USA) for 15 min, and absorbance was measured at 600 nm to evaluate biofilm formation [1,38]. All experiments were conducted in triplicate under identical conditions for reproducibility.

### 2.5. Detection of Antibiotic Resistance and QS-Realted Gene Expression

Gene expression was evaluated by PCR amplification using primers targeting antibiotic resistance genes (*tetA*, *tetD*, *tetE*, *tetM*, *tetB*) and QS-related genes (*ahyI*, *ahyR*, *litR*), as listed in Table 1. PCR conditions for resistance genes were: 95 °C for 3 min; 35 cycles of 95 °C for 30 s, 55 °C for 30 s, and 72 °C for 30 s; and a final extension at 72 °C for 5 min [39,40,41]. For QS-related genes: 95 °C for 3 min; 40 cycles of 95 °C for 30 s, 54 °C for 15 s, and 72 °C for 30 s; final extension at 72 °C for 10 min [42,43,44]. Amplified PCR products were electrophoresed on a 1.5% agarose gel, and target bands were purified using the QIAquick^®^ Gel Extraction Kit (Qiagen, Frankfurt, Germany) before sequencing. Sequences were identified using BLASTn (NCBI, accessed via the NCBI website). A no-template control (NTC) was included in each PCR run to confirm the absence of contamination and ensure amplification specificity.

### 2.6. qRT-PCR

Total RNA was extracted from antibiotic-treated bacterial cultures using the easy-spin Total RNA Extraction Kit (iNtRON Biotechnology, Seongnam, Korea) according to the manufacturer’s instructions. cDNA was synthesized using the iScript™ cDNA Synthesis Kit (Bio-Rad, Hercules, CA, USA). qRT-PCR was performed on a QuantStudio™ 5 Real-time PCR System (Applied Biosystems, Foster City, CA, USA) using PowerTrack™ SYBR™ Green Master Mix (Applied Biosystems, Foster City, CA, USA). The 20 μL PCR reaction mixture included 10 μL SYBR Green mix, 0.5 μL 40X Yellow sample buffer, 1 μL each of forward and reverse primers for *ahyI*, *ahyR*, *litR*, and *fleQ* (Table 1), 0.5 μL cDNA, and 7 μL nuclease-free water. The qRT-PCR cycling conditions were: 42 °C for 5 min; 95 °C for 2 min; followed by 45 cycles of 95 °C for 15 s, 55 °C for 20 s, and 72 °C for 20 s [45]. Melting curve analysis was performed to confirm specific amplification. Gene expression levels were calculated using the 2^−ΔΔCt^ (ΔΔCt = ΔCt target gene − ΔCt reference gene) method [46], and first derivative melting curves were generated using the QuantStudio™ Design and Analysis Software v1.0 [47].

### 2.7. Statistical Analysis

Statistical analyses were performed using one-way analysis of variance (ANOVA) to compare differences among multiple groups. Prior to ANOVA, the assumptions of normality and homogeneity of variance were tested using the Shapiro–Wilk test and Levene’s test, respectively. Tukey’s post hoc test was conducted to identify significant differences between group means. Additionally, independent *t*-tests (*p* < 0.05) were performed using SPSS v28.0 (SPSS Inc., Chicago, IL, USA) for pairwise comparisons where applicable. All data are presented as mean ± standard deviation (SD) from at least three independent experiments. Statistical significance was defined as *p* < 0.05. Graphs and statistical visualizations were generated using GraphPad Prism 9.0 (GraphPad Software, San Diego, CA, USA).

## 3. Results

### 3.1. Identification of Bacterial Isolates

**Figure 1:**
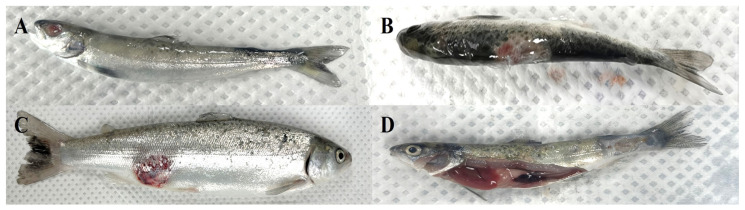
External clinical signs in Atlantic salmon (*Salmo salar*) infected with *Aeromonas salmonicida* subspecies. (**A**) Ulceration in the dorsal fin region and exophthalmia. (**B**) Dorsal fin necrosis and generalized skin darkening (melanosis). (**C**) Severe ulcerative lesion on the lateral body surface. (**D**) Pallor of the gills, splenomegaly, and skin ulceration accompanied by internal hemorrhage.

External symptoms including gill pallor, skin reddening and ulceration, fin hemorrhage and necrosis, skin darkening, and ocular loss were observed in a total of 67 Atlantic salmon collected in 2023 and 2024 (Figure 1). Among these fish, *Aeromonas salmonicida* was isolated from 25 individuals, with 21 bacterial strains identified based on 16S rRNA gene sequencing. Subspecies classification based on *vapA* gene sequence analysis revealed that 11 isolates belonged to *A. salmonicida* subsp. *masoucida* (ASM), while 10 isolates were identified as *A. salmonicida* subsp. *salmonicida* (ASS). The biochemical and molecular identification characteristics of these isolates have been described in detail in our previous study, “Characterization of *Aeromonas salmonicida* subsp. *masoucida* and subsp. *salmonicida* isolated from Atlantic salmon (*Salmo salar*)” [48]. For clarity and reproducibility, the criteria used to distinguish *A. salmonicida* from other accompanying microflora on TSA plates are described in the Materials and Methods Section 2 (Section 2.2 and Section 2.3).

### 3.2. Analysis of Growth Inhibition and Biofilm Formation

ASM (*n* = 11) and ASS (*n* = 10) strains were treated with four antibiotics-ampicillin, amoxicillin, oxytetracycline, and doxycycline-at various concentrations. Bacterial growth (OD_600_) and biofilm formation (biofilm OD_600_) were measured at 24, 48, 72, and 96 h post-treatment.

#### 3.2.1. *Growth Inhibition*

**Figure 2:**
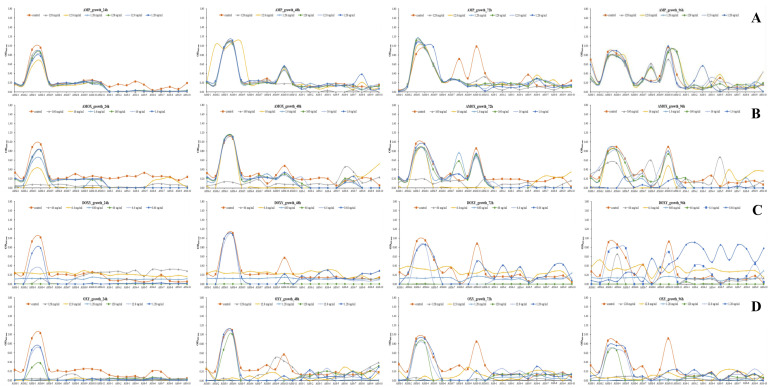
Growth kinetics of *Aeromonas salmonicida* subspecies strains in response to four antibiotics over time courses of 24, 48, 72, and 96 h. Bacterial growth was assessed by measuring optical density at 600 nm (OD_600_) across a range of antibiotic concentrations. Each graph represents individual responses of *A. salmonicida* subsp. *masoucida* (ASM) and *A. salmonicida* subsp. *salmonicida* (ASS) strains under control and treated conditions. (**A**) Ampicillin. (**B**) Amoxicillin. (**C**) Doxycycline. (**D**) Oxytetracycline. Data are presented as mean ± standard deviation (SD) from three independent experiments. Statistical significance was determined by one-way ANOVA followed by Tukey’s multiple comparison test. Asterisks indicate significant differences compared with the control group (*p* < 0.05, *p* < 0.01).

Significant growth inhibition was observed in both ASM and ASS strains at high antibiotic concentrations (>12.8 mg/mL, >16 mg/mL, >6.4 mg/mL), with a more pronounced effect in ASS strains (Figure 2). For example, after 24 h of treatment with 128 mg/mL ampicillin, the average OD_600_ of ASS was nearly undetectable (0.00045 ± 0.00039), whereas ASM maintained relatively higher growth (0.303 ± 0.248) (Figure 2A). Similarly, 160 mg/mL amoxicillin treatment resulted in lower growth in ASS (0.042 ± 0.03) compared to ASM (0.058 ± 0.013) (Figure 2B). Interestingly, under 64 mg/mL doxycycline treatment, ASM (0.094 ± 0.097) showed lower growth than ASS (0.283 ± 0.044), indicating higher sensitivity of ASM to doxycycline (Figure 2C). In the 128 mg/mL oxytetracycline group, the average OD_600_ was slightly lower in ASS (0.04 ± 0.017) than in ASM (0.054 ± 0.037), with marked growth inhibition observed in both subspecies (Figure 2D). In most high-concentration antibiotic groups, growth inhibition in ASS persisted until 96 h. In contrast, ASM exhibited a gradual increase in growth starting at 48 h under low-concentration conditions (<12.8 μg/mL, <16 μg/mL, <6.4 μg/mL), with some isolates recovering to control-level growth by 72–96 h.

#### 3.2.2. *Biofilm Formation*

**Figure 3:**
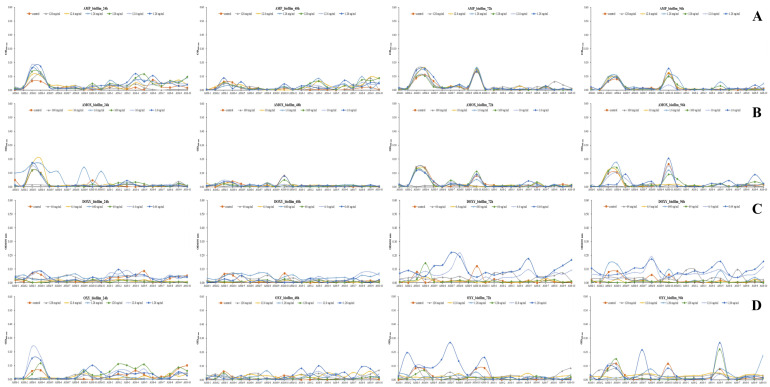
Biofilm formation by *Aeromonas salmonicida* subspecies strains in response to four difference antibiotics over a time course of 24, 48, 72, and 96 h. Biofilm biomass was quantified by measuring the optical density at 600 nm (OD_600_) after crystal violet staining across a range of antibiotic concentrations. Each graph displays the biofilm-forming responses of individual *A. salmoncidia* subsp. *masoucida* (ASM) and *A. salmonicida* subsp. *salmonicida* (ASS) strains under both control and treatment conditions. (**A**) Ampicillin. (**B**) Amoxicillin. (**C**) Doxycycline. (**D**) Oxytetracycline. Data are expressed as mean ± standard deviation (SD) of triplicate measurements. Statistical analysis was performed using one-way ANOVA followed by Tukey’s post hoc test to compare between groups. Asterisks denote significant differences compared with the control or between treatment groups (*p* < 0.05, *p* < 0.01).

Overall, biofilm formation was higher under low antibiotic concentrations than under high concentrations, with notable differences depending on the antibiotic type and bacterial subspecies. Biofilm formation generally peaked at 24–48 h and gradually declined after 72 h (Figure 3). ASM exhibited stronger and more sustained biofilm formation compared to ASS. At 24 h after treatment with 128 mg/mL ampicillin, the average biofilm OD_600_ of ASM and ASS was 0.036 ± 0.033 and 0.014 ± 0.012, respectively. Under 1.28 μg/mL treatment, these values increased to 0.044 ± 0.055 (ASM) and 0.046 ± 0.014 (ASS) (Figure 3A). For amoxicillin, high-concentration (160 mg/mL) treatment yielded low OD_600_ values (ASM: 0.009 ± 0.004, ASS: 0.012 ± 0.01), which increased under 1.6 μg/mL to 0.029 ± 0.052 (ASM) and 0.012 ± 0.011 (ASS), with a more marked increase observed in ASM (Figure 3B). With 64 mg/mL doxycycline, OD_600_ was 0.031 ± 0.013 (ASM) and 0.013 ± 0.008 (ASS), while at 0.64 μg/mL, it increased to 0.034 ± 0.024 (ASM) and 0.044 ± 0.023 (ASS), indicating a more pronounced change in ASS (Figure 3C). Oxytetracycline treatment (128 mg/mL) resulted in OD_600_ values of 0.013 ± 0.012 (ASM) and 0.014 ± 0.016 (ASS), which increased to 0.05 ± 0.053 and 0.033 ± 0.018, respectively, at 1.28 μg/mL (Figure 3D). These findings suggest that while high antibiotic concentrations suppress biofilm formation, sub-inhibitory concentrations may enhance it. This indicates that biofilm formation may serve as a bacterial survival strategy in response to antibiotic-induced stress.

### 3.3. Expression of Antibiotic Resistance and Quorum Sensing (QS) Genes

#### 3.3.1. Expression of Tetracycline Resistance Genes

**Figure 4:**
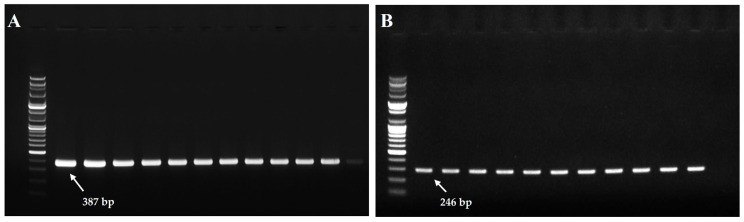
PCR amplification of tetracycline resistance genes in *Aeromonas salmonicida* subsp. *masoucida* strains. PCR products were separated by electrophoresis on a 1.5% agarose gel and visualized under UV transillumination. The expected amplicon sizes for *tetA* and *tetE* were 387 bp and 246 bp, respectively. A no-template control (NTC) was included to verify the absence of contamination. (**A**) Amplification of the *tetA* gene. (**B**) Amplification of the *tetE* gene.

PCR analysis revealed the presence of tetracycline resistance genes (*tetA*, *tetB*, *tetD*, *tetE*, *tetM*) in all ASM isolates (*n* = 11), with *tetA* and *tetE* consistently detected. In contrast, none of these resistance genes were detected in ASS isolates (*n* = 10) (Figure 4), suggesting a genetic basis for the higher tetracycline resistance observed in ASM.

#### 3.3.2. *Expression of QS-Related Genes*

**Figure 5:**
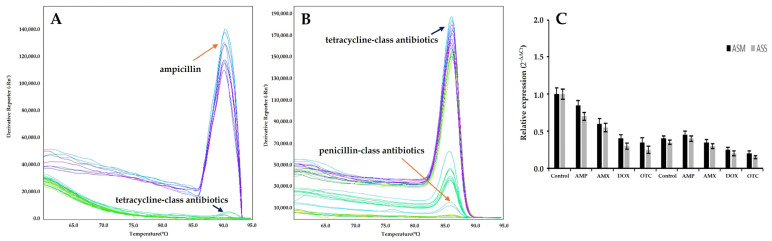
Melting curve analysis of quorum sensing-related gene expression in *Aeromonas salmonicida* subspecies strains, assessed by qRT-PCR. Melting curves were generated to verify the specificity of the qRT-PCR amplification. Each distinct peak corresponds to a specific PCR product, indicating the absence of primer-dimer artifacts or non-specific amplification. Derivative reporter fluorescence (-Rn) is plotted against temperature (°C). (**A**) Expression of the *ahyR* gene. (**B**) Expression of the *litR* gene. (**C**) Relative expression of the *ahyR* and *litR* genes in *A. salmonicida* subsp. *masoucida* and *A. salmonicida* subsp. *salmonicida* under β-lactam (ampicillin, amoxicillin) and tetracycline (doxycycline, oxytetracycline) antibiotic treatments. Relative expression levels were calculated using the 2^−ΔΔCt^ method with *fleQ* as the reference gene. Error bars represent standard deviation (SD) from three independent experiments.

To investigate the response of QS-related genes to antibiotic exposure, the expression levels of *ahyI*, *ahyR*, and *litR* were analyzed in ASM and ASS using qRT-PCR. Following treatment with tetracycline-class antibiotics such as oxytetracycline and doxycycline, expression of *ahyI* and *ahyR* was generally downregulated in both subspecies (Figure 5). However, in response to ampicillin, some ASS isolates (e.g., ASS-4, ASS-8) exhibited upregulated *ahyI* expression (Figure 5A). Expression of *ahyR* remained largely unchanged across all treatment groups. Expression of *litR*, known to function as a QS repressor, showed a differential response depending on the antibiotic. Notably, more than a twofold increase in *litR* expression was observed in ASM-2, ASM-7, ASS-5, and ASS-9 upon treatment with oxytetracycline and doxycycline (Figure 5B), suggesting that *litR* may be activated under antibiotic-induced stress. In contrast, under penicillin-class antibiotic treatment, *litR* expression remained largely unchanged or even decreased in some isolates, indicating a relatively limited impact on the QS repression pathway. To further visualize the overall trend of gene regulation, the relative expression levels of *ahyR* and *litR* were quantified using the 2^−ΔΔCt^ method and summarized in Figure 5C. Both genes exhibited markedly lower expression under tetracycline-class antibiotic exposure (doxycycline and oxytetracycline) compared with the control and β-lactam antibiotics (ampicillin and amoxicillin). The reduction was more pronounced in ASS than in ASM, indicating that *A. salmonicida* subsp. *salmonicida* is more susceptible to tetracycline-induced suppression of QS-related gene expression. These results collectively suggest that tetracycline stress may attenuate quorum-sensing signaling, potentially leading to reduced biofilm-forming ability in *A. salmonicida*.

## 4. Discussion

Bacteria form biofilms as a key survival strategy to protect themselves from external environmental stressors. This collective lifestyle enhances resistance to antibiotics and disinfectants [49]. Within biofilms, horizontal gene transfer is actively facilitated, enabling efficient acquisition and dissemination of resistance and virulence genes [50]. Consequently, major pathogens in aquaculture environments-such as *Vibrio* spp., *Flavobacterium* spp., and *Aeromonas* spp. -are recognized biofilm producers and have demonstrated resistance to a variety of antibiotics and disinfectants [38,51,52]. However, studies investigating the relationship between aquaculture antibiotics and biofilm formation remain limited [24,53].

In this study, we comparatively analyzed the growth dynamics, biofilm formation, expression of antibiotic resistance genes, and quorum sensing (QS)-related gene expression (*ahyI*, *ahyR*, *litR*) in *Aeromonas salmonicida* subsp. *masoucida* (ASM) and *A. salmonicida* subsp. *salmonicida* (ASS) under various antibiotic concentrations. The aim was to elucidate the interactions between antibiotic exposure, biofilm development, and gene regulation, with the goal of identifying alternative strategies for controlling pathogenicity beyond conventional antibiotic-based approaches.

Growth inhibition assays revealed that both ASM and ASS strains exhibited suppressed growth under high antibiotic concentrations, with ASS showing greater susceptibility, as evidenced by significantly lower OD_600_ values. This differential response likely reflects subspecies-specific differences in antibiotic resistance, potentially due to variations in genetic composition or phenotypic traits [28]. Resistance in *A. salmonicida* is often associated with plasmid-encoded genes [54]; however, functional resistance is governed by complex interactions involving gene expression, regulatory networks, and physiological adaptations [55]. Interestingly, some isolates exhibited growth recovery or maintained levels comparable to the control at sub-MIC concentrations, suggesting metabolic adaptation and inducible resistance mechanisms [6].

Biofilm assays demonstrated that sub-MIC antibiotic exposure significantly enhanced biofilm formation in both subspecies. Notably, ASM strains showed higher and more sustained biofilm production than ASS, indicating a more active biofilm-based survival strategy. These findings are consistent with previous reports showing that sub-MIC levels of antibiotics can stimulate biofilm formation [14,56], likely by limiting antibiotic diffusion and creating altered microenvironments within the biofilm that favor resistance [57]. However, a gradual decline in biofilm biomass was observed after 72–96 h of incubation. This reduction likely reflects the dispersal phase of the biofilm life cycle rather than simple growth inhibition. During the late stage of biofilm maturation, nutrient limitation and oxygen depletion within the deeper layers can induce cell death and detachment from the biofilm matrix. In addition, antibiotic-induced stress may trigger intracellular signaling pathways that promote biofilm dispersal and the release of planktonic cells. Such dispersal behavior has been reported as a regulated survival mechanism that allows bacteria to escape nutrient-depleted environments and colonize new surfaces [58,59]. Therefore, the decrease in biofilm biomass observed at 72–96 h may indicate a physiological transition from the maturation to the dispersal stage of the biofilm.

QS is a key regulatory mechanism that mediates bacterial communication and EPS production, playing a central role in biofilm development [45,60,61]. In *Aeromonas* spp., QS-particularly via *ahyI* and *ahyR*-is critical for the synthesis of *N*-acyl homoserine lactones (AHLs), which coordinate group behavior [62,63]. qRT-PCR analysis revealed significant downregulation of *ahyI* and *ahyR* in both subspecies following treatment with oxytetracycline and doxycycline. *ahyI* encodes an AHLs synthase, while *ahyR* encodes the corresponding receptor that activates QS-regulated gene expression [64,65]. Suppression of these genes under antibiotic stress suggests potential for disrupting bacterial virulence via QS interference [60,61]. The *litR* gene, a LuxR-family transcriptional repressor of QS, was downregulated in most antibiotic-treated strains, with some exceptions. This may indicate a transition to a quiescent or less virulent phenotype under antibiotic pressure [66]. However, in some isolates, increased *litR* expression coincided with elevated biofilm formation, suggesting that biofilm regulation may also be governed by other regulatory systems, including c-di-GMP signaling and transcription factors such as *fleQ* [30,31]. Interestingly, this paradoxical pattern—where increased *litR* expression coincides with enhanced biofilm formation—may be attributed to the multifaceted regulatory nature of *litR* and its interaction with other signaling systems. While *litR* is generally recognized as a QS repressor, recent studies have shown that its function can vary depending on environmental stress and cellular context. Under antibiotic-induced or sub-MIC stress conditions, *litR* may be involved in adaptive regulation through secondary messenger pathways such as cyclic-di-GMP, influencing EPS synthesis or cell-surface adhesion. Therefore, the observed co-occurrence of elevated *litR* expression and increased biofilm biomass likely reflects a compensatory stress response rather than a canonical QS repression. This suggests that biofilm formation in *Aeromonas salmonicida* is governed by an integrated network that coordinates QS, stress signaling, and metabolic regulation rather than a single gene function.

Expression of tetracycline resistance genes further highlighted subspecies-specific differences: *tetA* and *tetE* were consistently detected in ASM isolates, whereas no resistance genes were identified in ASS. Although PCR analysis confirmed the presence of antibiotic resistance genes, this method is qualitative and does not provide information regarding their transcriptional expression levels. Therefore, the detection results should be interpreted as indicative of potential resistance rather than actual gene activity. To better understand the functional expression and regulatory mechanisms of these genes under antibiotic stress, future studies should employ quantitative approaches such as qRT-PCR or transcriptomic analyses (e.g., RNA-seq). This finding underscores that antibiotic resistance is not solely determined by gene presence but also by functional expression, biofilm-associated protection, membrane permeability, and interactions with QS networks [55].

Overall, this study elucidates the complex interplay among antibiotic response, biofilm formation, and QS regulation in aquaculture pathogens. The results highlight the potential of targeting sub-MIC effects, QS systems, and biofilm physiology to develop precision-based strategies for pathogen control. These insights contribute to the optimization of antibiotic use in aquaculture and inform future efforts to combat functional resistance. However, given that this study was conducted under in vitro conditions, further in situ validation is required to assess the practical applicability of these findings. Future research should investigate integrated regulatory pathways, including c-di-GMP signaling, additional transcriptional regulators, and environmental stimuli, to advance our understanding and control of biofilm-associated pathogenicity.

## 5. Conclusions

This study investigated the effects of antibiotic exposure on growth dynamics, biofilm formation, and gene expression in *Aeromonas salmonicida* subspecies isolated from Atlantic salmon. Both *A. salmonicida* subsp. *masoucida* (ASM) and *A. salmonicida* subsp. *salmonicida* (ASS) exhibited distinct responses under varying antibiotic concentrations, with ASM demonstrating greater biofilm formation and tolerance at sub-MIC levels. Exposure to sublethal antibiotic concentrations markedly enhanced biofilm development, suggesting that such conditions may promote bacterial persistence in aquaculture environments. Downregulation of quorum sensing (QS)-related genes (*ahyI*, *ahyR*, and *litR*) under antibiotic stress indicates that interference with QS systems could serve as an alternative strategy to mitigate virulence. Furthermore, the presence of *tetA* and *tetE* in ASM, but not in ASS, highlights subspecies-specific resistance mechanisms. Overall, these findings reveal a complex interplay among antibiotics, biofilm physiology, and QS regulation, emphasizing the importance of considering sub-MIC effects in aquaculture antibiotic management. Future in situ investigations are warranted to validate these mechanisms and to develop precise, non-antibiotic approaches for controlling *A. salmonicida* infections in farmed salmon.

## Figures and Tables

**Table 1:** Primer sequences, expected amplicon size, and literature references for target genes used in quantitative real-time PCR and quantitative PCR analyses.

Primer	Sequence (5′-3′)	Amplicon Size (bp)	References
*TetA*	F-GCGCTNTATGCGTTGATGCA	387	[40]
	R-ACAGCCCGTCAGGAAATT		
*TetD*	F-GCGCTNTATGCGTTGATGCA	484	
	R-CCAGAGGTTTAAGCAGTGT		
*TetE*	F-GCGCTNTATGCGTTGATGCA	246	
	R-ATGTGTCCTGGATTCCT		
*TetM*	F-GTGGACAAAGGTACAACGAG	406	[41]
	R-CGGTAAAGTTCGTCACACAC		
*TetB*	F-CTCAGTATTCCAAGCCTTTG	416	[39]
	R-CTAAGCACTTGTCTCCTGTT		
*ahyI*	F-GTCAGCTCCCACACGTCGTT	202	[42]
	R-GGGATGTGGAATCCCACCGT		
*ahyR*	F-TTTACGGGTGACCTGATTGAG	206	[43]
	R-CCTGGATGTCCAACTACATCTT		
*litR*	F-CATCGAGGTGTTCTCCCGTC	123	[44]
	R-TCATCCACCAGCTCTTCACG		

## Data Availability

The original contributions presented in this study are included in the article. Further inquiries can be directed to the corresponding author.
